# Pilot study investigating BP-180 in extracellular vesicles derived from blister fluid of bullous pemphigoid patients

**DOI:** 10.1007/s00403-023-02560-2

**Published:** 2023-02-10

**Authors:** Giulia Gasparini, Roberta Tasso, Maria Elisabetta Federica Palamà, Maria Chiara Ciferri, Chiara Gentili, Giovanni Di Zenzo, Alessia Provini, Adele Salemme, Rodolfo Quarto, Aurora Parodi, Emanuele Cozzani

**Affiliations:** 1 Secction of Dermatology, Department of Health Sciences (DISSAL), University of Genoa, Genoa, Italy; 2 Dermatology Unit, Ospedale Policlinico San Martino IRCCS, Genoa, Italy; 3 Department of Experimental Medicine (DIMES), University of Genoa, Genoa, Italy; 4 Molecular and Cell Biology Laboratory, IDI-IRCCS, Rome, Italy; 5 Dermaology Unit IDI-IRCCS, Rome, Italy; 6 UO Cellular Oncology, IRCCS Ospedale Policlinico San Martino, Genoa, Italy

**Keywords:** Bullous pemphigoid, Extracellular vescicles, Exosomes, BP180

## Abstract

Bullous pemphigoid (BP) is an autoimmune blistering disease that targets the haemidesmosomal proteins, mainly BP180. Extracellular vesicles (EVs) have been demonstrated to carry tissue-specific autoantigens in the setting of autoimmune diseases and transplant organ rejection; this phenomenon was demonstrated to have pathogenic implications in autoimmune diseases and to correlate with transplant rejection severity. The purpose of this study was to identify the presence of BP targeted autoantigens in blister fluid derived EVs. We isolated, by size exclusion chromatography, EVs derived from blisters of BP-patients and from suction blisters of healthy donors. EV characterization was performed by flow cytometry and nanoparticle tracking analysis. Western blot analysis was used to investigate the presence of autoantigens. A suspension enriched in EVs was efficiently obtained from blister fluid from patients and healthy donors. EV-enriched fractions were enriched in particles with a size distribution characterizing small-EVs (main peak was present at 94.5 nm). BP180 was found, by western blot analysis, in EVs derived from blister fluid of 3 out 6 BP patients and in none of EVs isolated from suction blister fluid of healthy donors. BP230 and Dsg1 were not detectable in EVs of any of the samples. No specific clinical characteristics seemed to correlate to the presence of BP180 in EVs. The discovery of BP180 in EVs derived from blister fluid might help understanding BP pathogenesis.

**Supplementary Information:**

The online version contains supplementary material available at 10.1007/s00403-023-02560-2.

## Introduction

Bullous pemphigoid (BP) is an organ-specific autoimmune blistering disease, mediated by autoantibodies directed against haemidesmosomal proteins, mainly BP180 [1].

Extracellular vesicles (EVs) are small membranous structures that encapsulate a variegate cargo (proteins, miRNA, mRNA and lipids) and mediate intercellular communication [2]. Based on size, origin and function, EVs are classified into exosomes (40–160 nm), microvesicles (100–1000 nm) and apoptotic bodies (50–5000 nm) [3, 4]. Recently, EVs have been demonstrated to carry tissue-specific autoantigens in autoimmune diseases [2]. Indeed, EVs can transport pancreatic inslet antigen 2 (IA2), anti-glutamate acid decarboxylase (GAD65), proinsulin and insulin in type 1 diabetes mellitus [5, 6] and citrullinated proteins in rheumatoid arthritis [7]. Moreover, in organ transplant rejection, circulating EVs containing tissue-antigens related to the failing organ can be found [8, 9]. These phenomena were demonstrated to have pathogenic implications in autoimmune diseases and to correlate with transplant rejection severity.

The aim of our study was to identify the presence of BP targeted autoantigens in blister fluid derived EVs (BF-EVs) in BP patients.

## Materials and methods

All patients had recently diagnosed active forms of BP, confirmed by characteristic histological and immunological findings. Study population included 9 BP patients with idiopathic BP and 2 patients with drug-induced BP and 6 healthy controls (3 suction blisters, 2 burn blisters and 1 pressure blister). Suction blisters on the volar surface of forearms were obtained by Hijama cups [10]. Blister fluid was centrifugated twice at 4 °C at 2500RPM for 10 min, frozen and stocked at − 20 °C. The study was approved by the local Ethical Review Board (Comitato Etico Regione Liguria: N. Registro CER Liguria: 12/2021—DB id 11,151).

EVs were isolated from BF by size-exclusion chromatography (SEC) with qEV70 columns (Izon Science, Christchurch, New Zealand), that contain a resin with approximately 70 nm pore size [11]. Prior SEC columns were washed with 15 ml of freshly 0.2 μm filtered phosphate-buffered saline (PBS). Thawed fluid (500 µl) was added to the sample reservoir and EVs were eluted in PBS, which was added to the sample reservoir as the last of the fluid entered the column. The initial six fractions of flow-through were discarded, EVs were collected in the pooled fractions 7–9 using Protein LoBind tubes, while contaminating proteins were eluted from fraction 10 to fraction 22. The elution profile of both EVs and contaminating proteins has been assessed measuring the absorbance at 280 nm.

EV characterization by flow cytometry was performed as previously described [12]. For each preparation, one tube was stained with 1 μM CFDA-SE at 4 °C (Vybrant™ CFDA-SE Cell Tracer Kit, ThermoFisher Scientific) as control, and one tube containing EVs was stained with the same amount of CFDA-SE at RT to visualize intact EVs and set the correct dimensional gate. A mixture of fluorescent beads of varying diameters (Megamix-Plus FSC and Megamix-Plus SSC, Biocytex) was used to discriminate EV size. Expression of EV markers CD9 (APC Mouse Anti-Human CD9, Biolegend, 312,108), CD63 (PE-Cy7 Mouse Anti-Human CD63, BD Horizon, 561,982), CD81 (BV421 Mouse Anti-Human CD81, BD Biosciences, 740,079) and the corresponding isotype controls APC Mouse IgG1, κ Isotype Ctrl (FC) Antibody (Clone MOPC-21, Biolegend), PE-Cy7 Mouse IgG1, κ Isotype Ctrl (FC) Antibody (555,872, BD Horizon), and BV421 Mouse IgG1, κ Isotype Ctrl Antibody (562,438, BD Biosciences), were evaluated within the CFDA-SE-positive events using the BD FACSAriaII (BD Biosciences).

For western blot analysis EVs were resuspended in RIPA buffer (1% NONIDET p-40, 0.1% SDS, 0.1% Sodium deoxycholate, protease inhibitor cocktail 1x, in PBS pH7.5) and protein content was quantified by Bicinchoninic acid (BCA) assay (Thermo Scientific, Waltham, MA USA). Two μg of proteins for each sample, were loaded on 4%–12% NuPAGE Bis–Tris gel (Life Technologies, Carlsbad, California, USA). Electrophoresis was performed at 150 V and proteins were blotted on a polyvinylidene fluoride membrane (Millipore, Burlington, Massachusetts, USA). After blocking nonspecific sites with 5% non-fat dry milk (EuroClone, Italy) in Tris Buffered Saline with Tween 20 (TTBS, 20 mM Tris pH 7.5, 500 mM NaCl, 0.05% Tween 20), blot membrane was incubated overnight at 4 °C with specific primary antibodies for: rabbit anti-human BP180 (1:1000, in house), goat anti-human BP230 (1:40, Santa-Cruz) goat anti-human DSG1 (1:1000, R&D), and mouse anti-human CD63 (1:1000 dilution, 10628D, Invitrogen), prepared in 2.5% non-fat dry milk/TTBS. After three washes with TTBS, membranes were incubated with a specific HRP-conjugated secondary antibody (1:2000 dilution, Cell Signaling Technology, Danvers, Massachusetts, USA). Positivity was highlighted by providing the substrates for the chemiluminescence reaction of HRP (Amersham ECL Prime Western Blotting Detection Reagent, GE Healthcare, Chicago, Illinois, USA) and impressing a photographic sheet by autoradiography (GE Healthcare). Images were scanned using the Epson perfection 1260 scanner. Gel running was performed under not denatured conditions for the detection of CD63 and under denatured conditions for other markers.

## Results

Demographic and clinical characteristics of patients and controls are summarized in Table 1.

**Table 1:** Demographics, clinical and immunological characteristics and western blot results of BP patients and controls

Pt ID	Age	Gender	Drug induced	Blister type	Severity	BPDAI	New diagnosis	BF collection since BP onset (months)	Immuno-suppressive therapy at collection time	ELISA IgG anti-BP180-NC16A	ELISA IgG anti-BP230	IIF/IIF-SSS	DIF	WB EVs 180 kDa	WB EVs 230 kDa	WB EVs Dsg1
BP1	77	F	Yes gliptine	Bullous pemphigoid	Mild	8	Yes	1	No	> 200	30	Nd	Nd	Neg	Neg	Neg
BP2	70	F	No	Bullous pemphigoid	Moderate	30	Yes	1	Clobetasol 0,05% cream qd	> 200	Neg	Nd	Linear IgG/C3 DEJ	Neg	Neg	Neg
BP3	78	M	Yes nivolumab	Bullous pemphigoid	Mild	8	No	7	Prednisone 25 mg qd	104	180	Nd	Nd	Pos	Neg	Neg
BP4	80	F	No	Bullous pemphigoid	Moderate	25	No	3	Prednisone 37,5 mg qd, azathioprine 50 mg qd	> 200	Neg	Nd	Nd	Pos	Neg	Neg
BP5	84	M	No	Bullous pemphigoid	Severe	62	Yes	1	No	164	Neg	Nd	Linear C3 DEJ	Neg	Neg	Neg
BP6	66	F	No	Bullous pemphigoid	Severe	67	Yes	1	No	80	Neg	Nd	Linear C3 DEJ	Pos	Neg	Neg
BP7	74	M	No	Bullous pemphigoid	Moderate	54	Yes	1	Prednisone 25 mg qd	136	124	Pos roof	Nd	Pos	Neg	Nd
BP8	85	M	No	Bullous pemphigoid	Moderate	42	Yes	3	Clobetasol 0,05% cream qd	91	10	Nd	Nd	Pos	Neg	Nd
BP9	69	F	No	Bullous pemphigoid	Moderate	25	Yes	1	No	Neg	Neg	Linear IgG/C3 DEJ	Nd	Neg	Neg	Nd
BP10	82	M	No	Bullous pemphigoid	Mild	3	Yes	1	No	Neg	Neg	Neg	Linear IgG/C3 DEJ	Neg	Neg	Nd
BP11	82	M	No	Bullous pemphigoid	Mild	5	Yes	1	No	Neg	Neg	Linear IgG/C3 DEJ	Linear IgG/C3 DEJ	Neg	Neg	Nd
CTR1	60	M	–	Suction blister	–	–	–	–	–	–	–	–	–	Neg	Neg	Neg
CTR2	71	M	–	Suction blister	–	–	–	–	–	–	–	–	–	Neg	Neg	Neg
CTR3	36	M	–	Suction blister	–	–	–	–	–	–	–	–	–	Neg	Neg	Nd
CTR4	52	M	–	Burn	–	–	–	–	–	–	–	–	–	Neg	Neg	Nd
CTR5	40	F	–	Burn	–	–	–	–	–	–	–	–	–	Neg	Neg	Nd
CTR6	47	F	–	Pressure blister	–	–	–	–	–	–	–	–	–	Neg	Neg	Nd

*BF* blister fluid, *BPDAI* bullous pemphigoid disease area index

A suspension enriched in EVs was efficiently obtained from blister fluid of patients and healthy controls. From an average amount of 677 ml of patient-derived blister fluid and 532 ml of healthy control-blister fluid we were able to purify 25 mg and 19 mg of EVs, respectively, as measured by BCA assay. Nanoparticle tracking analysis indicated that the suspension was enriched in particles with a size distribution characterizing small-EVs (exosomes). The main peak was present at 94.5 nm, and a median size distribution of 123.9 nm, while the concentration of vesicles retrieved from 250 µl was 6.5 × 10^10^ particles/ml (Fig. S1a). At flowcytometry analysis, CFDA-SE stained particles under the level of the dimensional gate in the FL1 intensity channel expressed high levels of both CD81 and CD9 (84.6% and 84.2%, respectively), while the expression of CD63 was lower (5.6%), indicating that the suspension collected from the SEC column was mainly composed by EVs (Fig. S1b).

**Fig. 1:**
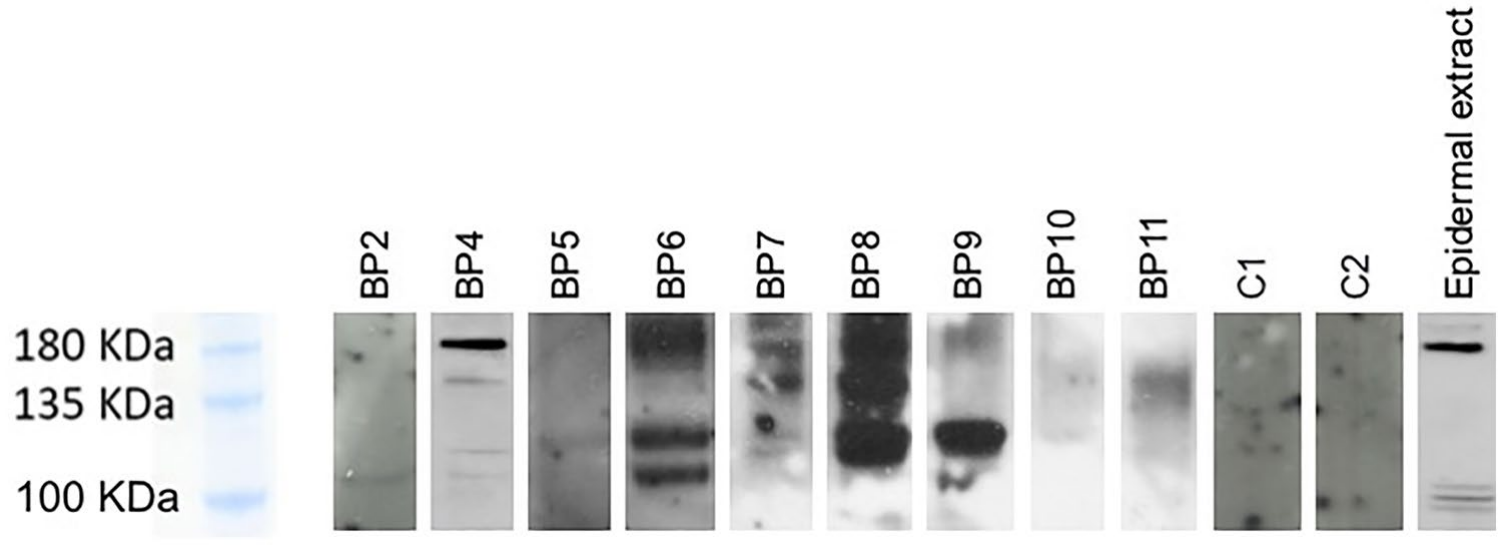
Western blot results. Detection of BP180 on blister fluid EVs derived from BP patients and healthy controls and on epidermic extract

For what concerns the idiopathic BP cases, a band at 180 kDa was detectable at variable intensity in 5 out of 9 patients (BP4, BP6, BP7, BP8, BP9). A band at 120KDa was clearly visible in 3 cases (BP6, BP8, BP9) and a faint band was detectable in 3 cases (BP4, BP5, BP7). Bands at a molecular weight of approximately 150KDa were visible in four cases (BP4, BP7, BP8, BP11) (Fig. 1). Regarding the drug-induced BP, a band at 180KDa was detected in BP3 and a band at circa 150KDa was present in patient BP1 (Fig. S2). Neither BP180 nor its cleaved forms were expressed by EVs from the blisters of healthy controls (Fig. 1). BP230 and Dsg1 were not detectable in any blister fluid (Fig. 1).

## Discussion

Our preliminary study was able to identify for the first time full length BP180 in BF-EVs in approximately half of cases. In some cases, bands at 120 kDa or circa 150 kDa were detected. These proteins might correspond to cleaved forms of BP180. Many variables likely contribute to the presence of full length BP180 and/or of its fragments in BF-EVs. Possibly, finding full-length BP180 in BF-EVs could be related to the stage of blister formation. Intact BP180 might be present in EVs immediately after blister formation but over time it might be cleaved by proteolytic enzymes into smaller fractions of 120 kDa or 150 kDa, and then it could be gradually degraded, until it cannot be found in EVs anymore. The presence of BP180 in BF-EVs did not seem to correlate with any specific clinical characteristics. No differences were observed between newly diagnosed patients or patients experiencing flares, nor between patient on immunosuppressive therapy or not.

BP230 was not detected in EVs, not even in the 2 patients with circulating anti-BP230 antibodies. Indeed, BP230 is a marginal targeted antigen in BP [13].

Dsg1 was considered as a negative control, as it is not a disease specific targeted autoantigen but at the same time it is of keratinocytic derivation, therefore its absence in BP-EVs supports disease specific release of BP180, rather than a generic antigen release from damaged keratocytes.

EV-encapsulated autoantigens in BP might represent a direct or indirect route of antigen presentation without cell-to-cell contact, a depletion route of BP180 contributing to the mechanical detachment of keratinocytes during blister formation, a way to spread among adjacent keratinocytes signals of internalization or depletion of BP180, or a prognostic marker of disease severity.

This is a pilot study, but the discovery of BP180, the most important autoantigen target in BP, in EVs derived from blister fluid might contribute to the understanding of BP pathogenesis.

## Supplementary Information

Below is the link to the electronic supplementary material.

Supplementary file1 Blister-fluid-derived EV characterization (a) Representative size distribution for blister fluid-EVs, analysed by Zetaview NTA. (b) Representative bidimensional dot plots (FL1-H vs. SSC-H, in logarithmic scale) for CFDA-SE specificity: EVs stained with CFDA-SE at 4°C (left panel) and EVs stained with CFDA-SE at room temperature (RT) (right panel). (C) Flow cytometry analysis of blister fluid-EVs. Areas under the black lines identify vesicles reacting with CD81 (left panel), CD63 (middle panel), and CD9 (right panel). Areas under the gray lines indicate the interactions of vesicles with corresponding non-reactive immunoglobulin of the same isotype (JPG 7 KB)

Supplementary file2 Western Blot results of Drug-induced BP (PNG 142 KB)
