# Supplementary figures and images for: Pilot study investigating BP-180 in extracellular vesicles derived from blister fluid of bullous pemphigoid patients
Source: Arch Dermatol Res. 2023 Feb 10;315(6):1837–41. doi: 10.1007/s00403-023-02560-2 (PMC10338600; doi:10.1007/s00403-023-02560-2)

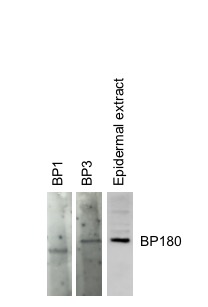

Supplement: Supplementary file 1 — Supplementary file1 Blister-fluid-derived EV characterization (a) Representative size distribution for blister fluid-EVs, analysed by Zetaview NTA. (b) Representative bidimensional dot plots (FL1-H vs. SSC-H, in logarithmic scale) for CFDA-SE specificity: EVs stained with CFDA-SE at 4°C (left panel) and EVs stained with CFDA-SE at room temperature (RT) (right panel). (C) Flow cytometry analysis of blister fluid-EVs. Areas under the black lines identify vesicles reacting with CD81 (left panel), CD63 (middle panel), and CD9 (right panel). Areas under the gray lines indicate the interactions of vesicles with corresponding non-reactive immunoglobulin of the same isotype (JPG 7 KB) [file 403_2023_2560_MOESM1_ESM.jpg]

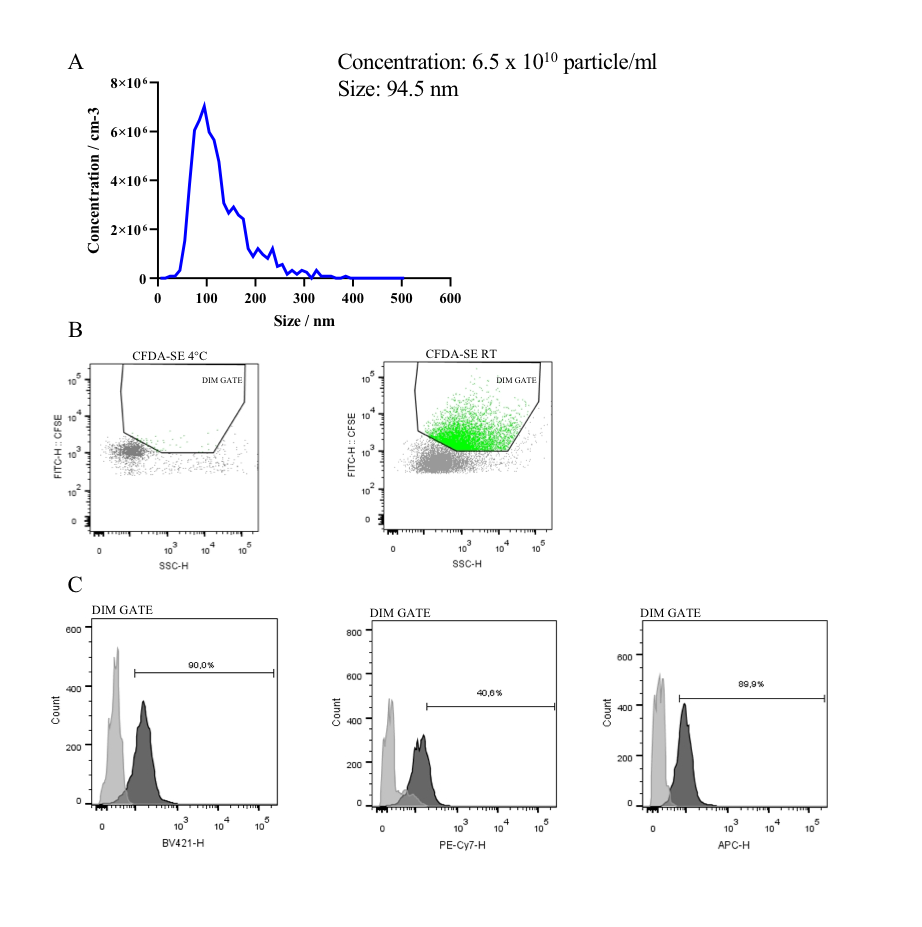

Supplement: Supplementary file 2 — Supplementary file2 Western Blot results of Drug-induced BP (PNG 142 KB) [file 403_2023_2560_MOESM2_ESM.png]
